# Genetic Dissection of Complex Genetic Factor Involved in NIDDM of OLETF Rat

**DOI:** 10.1155/2012/582546

**Published:** 2012-10-15

**Authors:** Takahisa Yamada, Hiroyuki Kose, Takeshi Ohta, Kozo Matsumoto

**Affiliations:** ^1^Laboratory of Animal Genetics, Division of Life and Food Sciences, Graduate School of Science and Technology, Niigata University, Nishi-ku, Niigata 950-2181, Japan; ^2^Division of Natural Sciences, Department of Life Science, International Christian University, Mitaka, Tokyo 181-8585, Japan; ^3^Biological/Pharmacological Research Laboratories, Central Pharmaceutical Research Institute, Japan Tobacco Inc., Takatsuki, Osaka 569-1125, Japan; ^4^Department of Laboratory Animal Science, Faculty of Integrated Life Sciences, Kyoto Sangyo University, Kita-ku, Kyoto 603-8555, Japan

## Abstract

The Otsuka Long-Evans Tokushima Fatty (OLETF) rat is an animal model for obese-type, noninsulin-dependent diabetes mellitus (NIDDM) in humans. NIDDM in this rat model was shown to be regulated by multiple genes. We have identified 14 quantitative trait loci (QTLs) responsible for NIDDM (*Nidd1-14/of*) on chromosomes 1, 5, 7, 8, 9, 11, 12, 14, 16, and 17 by a whole genome search in 160 F2 progenies obtained by mating the OLETF and the F344 rats. Among these loci, two QTLs, *Nidd1* and *2/of*, were declared significant loci at a genome-wide level. *Nidd3, 8, 9,* and *13/of* exhibited heterosis: heterozygotes showing significantly higher glucose levels than OLETF or F344 homozygotes. We also found evidence for interaction (epistasis) between *Nidd1/of* and *Nidd2/of*, between *Nidd1/of* and *Nidd10/of*, between *Nidd2/of* and *Nidd8/of*, and between *Nidd2/of* and *Nidd14/of*. Furthermore, *Nidd6* and *11/of* showed linkage with body weight, and *Nidd1, 2, 8, 9, 10,* and *12/of* had an interaction with body weight. These indicated that NIDDM in the OLETF would have a higher degree of genetic complexity. We suggest several interesting candidate genes located in rat genomic regions for *Nidd1-14/of* or the syntenic regions in human genome.

## 1. Introduction

Noninsulin-dependent diabetes mellitus (NIDDM), which represents the most common type of diabetes, is a major public health issue due to its increasing prevalence in many countries and the severity of its secondary complications [1]. Genetic predisposition is a major risk component of NIDDM as represented by numerous studies showing higher concordance rates in monozygotic than dizygotic twins [2, 3], higher incidence in offspring of diabetic parents [4], and correlation between risk of diabetes and ethnic ancestry in admixed populations in a common environment [5, 6]. Thus, identification of the genes involved in the expression of this disease would likely assist in the development of prevention and treatment strategies. However, with the exception of relatively rare early-onset forms that show autosomal dominant or mitochondrial inheritance [7], the genetics of human NIDDM is complex. Multiple genes contribute to the trait, and it is likely that there is significant genetic heterogeneity with environmental and lifestyle factors interacting with genetic predisposition [8, 9]. The obesity factor is likely to be of particular importance for NIDDM development [1, 10–12]. Additionally, genetic dissection of NIDDM is also confounded by incomplete penetrance. This complex disease process makes identification of the genes contributing to NIDDM in human populations quite difficult.

 An alternative approach to genetic studies in humans is to use an animal model with a similar phenotype to identify genetic mechanisms and then to use this information in directed studies of humans. Experimental animal models for complex human traits provide a means for circumventing two factors—heterogeneity and environment—that complicate human studies of complex traits, and experimental genetic crosses with inbred strains greatly simplify the analysis and interpretation of genotypic and phenotypic data. Therefore, studying the animal models may give us an opportunity for identifying the potential genetic factors controlling NIDDM development.

 The responsible loci for diabetes have been identified using a particular animal model. In the nonobese diabetic mouse, at least 15 different loci involved in insulin-dependent (type I) diabetes mellitus susceptibility have been mapped [13, 14], and studies to identify these genes are undertaken with congenic strategy and/or the potential candidate gene approach. Genetic analysis using the BB rat revealed that at least three genes are involved in the development of insulin-dependent diabetes mellitus: *Lyp *(*Iddm1*), which controls T cell lymphopenia, an MHC-linked gene (*Iddm2*), and the third gene (*Iddm3*) [15–17]. Very recently, a major insulin-dependent diabetes mellitus susceptibility gene, termed *Iddm/kdp1*, was identified on rat chromosome 11, in addition to an MHC-linked gene previously mapped, in the Long-Evans Tokushima Lean rat [18]. The first genome-wide quantitative trait locus (QTL) analysis in NIDDM has been reported by two independent laboratories [19, 20], which used the Goto-Kakizaki (GK) rat as an animal model. The GK rat is one of the best characterized animal models for genetic susceptibility to NIDDM in nonobese individuals [21–23]. The genetic dissection of NIDDM using this model has allowed to map at least seven loci involved in the disease on chromosomes 1, 2, 4, 5, 8, 10, and 17. However some characteristics of human NIDDM are not fully covered by the GK rat. Obesity as a major risk factor for NIDDM in humans has been known for 35 years, and in many patients NIDDM can be largely controlled by weight reduction [10, 24, 25]. The GK rat model does not exhibit obesity, and thus understanding of obese-type NIDDM in humans necessitates other animal models with characteristics of obesity. 

 The Otsuka Long-Evans Tokushima Fatty (OLETF) rat has been established by selective breeding based on impaired glucose tolerance from a spontaneously diabetic rat with polyuria, polydipsia, and mild obesity which was discovered in 1984 in an outbred colony of Long-Evans rats [26, 27]. The OLETF rat develops late onset hyperglycemia, a chronic course of disease, mild obesity, hyperplastic foci of pancreatic islets, and renal complication and has been considered as one of the best models for human NIDDM with mild obesity [27]. Prevention of obesity by exercise training [28] or a calorie-restricted diet [29] can prevent the development of NIDDM almost completely in the OLETF rat as in many humans with NIDDM, indicating that obesity is an important risk factor for NIDDM development in this rat. Therefore, the OLETF rat model is suitable for elucidating the genetic factor of obese-type NIDDM. 

## 2. Inheritance of NIDDM and Increase in Body Weight

We first examined inheritance of NIDDM and increase in body weight in the OLETF rat [30]. We crossed female OLETF rats with male nondiabetic Fischer-344 (F344) rats to generate F_1_ progeny which, in turn, were used to generate 160 male F_2_ progeny. Only males, 30 weeks of age, from the parental strains, the 28 F_1_ progeny and the 160 F_2_ progeny were phenotyped using the same protocol for oral glucose tolerance test (OGTT) and measurements of body weight. The glucose levels and body weight were increased in the OLETF rat as compared to the F344 rat, and the OLETF and F344 rats showed clear differences in all phenotypes (*P* < 0.0001). The F_1_ progeny showed completely different modes of inheritance for hyperglycemia and an increase in body weight. Hyperglycemia was inherited in an incompletely recessive manner, while an increase in body weight was inherited in an incompletely dominant manner. The different inheritance patterns suggest that NIDDM and body weight may be under different genetic control. Compared to the F_1_ progeny, the F_2_ progeny showed similar means but larger variance in all phenotypes. The broad, continuous distributions of glucose levels and body weight without evidence of distinct groups in each phenotype among the F_2_ progeny suggest that these traits are polygenically regulated.

 We estimated the proportion of genetic variance and the number of genes responsible for the traits [30]. For the glucose levels, the proportion of genetic variance in the F_2_ was found to be about 80–85% by comparison of the variances in F_1_ and F_2_ progenies, and the effective number of genes was estimated as two to seven by Wright's formula [31], depending upon the time of measurements. Roughly 60% of the F_2_ variance in body weight appeared to be genetic and the effective number of genes was estimated to be three. These results indicate that NIDDM and body weight are clearly polygenic and suggest that all phenotypes are amenable to genetic dissection.

## 3. Relationship between NIDDM and Body Weight

A linear regression analysis was used to investigate the relationship between body weight and the level of glucose in the parental strains, F_1_ animals and F_2_ animals [30]. The OLETF rat had higher correlations between body weight and levels of glucose, as compared to those of the F344 rat, indicating the integral relationship between body weight and NIDDM in the OLETF rat. The F_1_ animals showed the correlations of a degree similar to those of the OLETF rat. In the F_2_ progeny, there remained a strong positive correlation between body weight and the glucose levels (*r* = 0.483–0.569, *P* < 0.0001), indicating that the body weight accounts for a significant portion of the variation in glucose levels. This significant correlation is consistent with the role of obesity as an important factor in NIDDM development in the OLETF rat. Importantly, the correlations, on average, were lower in the F_2_ progeny than the F_1_ progeny suggesting the segregation of separate loci for body weight and NIDDM.

## 4. Identification and Characterization of NIDDM QTL

To identify the QTL(s) affecting susceptibility to NIDDM, we carried out a total genome scan on the F_2_ progeny using a set of 361 informative simple sequence length polymorphisms between the OLETF and F344 rats [30, 32, 33]. The markers were on average 4.7 cM apart. Overall 95.6 and 100% of the genome were within 10 and 20 cM, respectively, of an informative marker. Phenotypic and genotypic data were analyzed for linkage with the MAPMAKER/QTL computer package which is applied for the internal mapping based on single-QTL models [34–37]. We found statistically significant evidence for two QTLs affecting postprandial glucose levels in OGTT, that met the stringent criteria by Lander and Kruglyak [38] (Table 1). A gene on chromosome 7, near *D7Wox6*, showed a maximum LOD score of 4.33 for glucose levels 60 min after the glucose challenge and was designated *Nidd1/of* [30]. This locus accounted for 11.7% of the genetic variation (single gene model) in the F_2_ progeny. Another gene on chromosome 14, near *D14Rat3*, was linked (LOD score = 5.10) to the AUC for the entire glucose challenge and was designated *Nidd2/of*, accounting for 14.0% of the genetic variation (single gene model) in the F_2_ progeny [30]. 

*Nidd1/of* and *Nidd2/of* exerted effects on glucose levels at all time points after glucose challenge, and the inheritance pattern at the loci was consistent with OLETF alleles acting in a recessive or additive mode of inheritance to increase the level of plasma glucose. Significant interaction (epistasis) was found between these two loci (*P* = 0.0007 for glucose levels at 60 min, *P* = 0.0094 for glucose levels in AUC). The effect of the OLETF allele at one locus on glucose levels was maximal in animals homozygous for the OLETF allele at the other locus.

 Evidence for additional “suggestive” QTLs was found [30] (Table 1). *Nidd3/of*, linked to *D8Mgh9* on chromosome 8 (LOD score = 3.73), designates a suggestive locus for the level of glucose 30 min after the challenge. Interestingly, this locus exhibited heterosis: heterozygotes showing significantly higher glucose levels than OLETF homozygotes or F344 homozygotes, and no difference in effects between the two homozygote classes, reflecting an intra-allelic interaction. The region between *D11Mgh2* and *D11Mgh3* on chromosome 11 (*Nidd4/of*) was linked to fasting glucose levels (LOD score = 3.61). The OLETF alleles at *Nidd4/of* were associated with increased fasting glucose levels through acting in a dominant or additive manner. 

 In addition to *Nidd3* and *4/of*, we detected suggestive linkage of the trait of glucose levels to markers on chromosomes 1, 5, 7, 9, 12, 14, 16, and 17 [32] (Table 1). There existed evidence for two QTLs on chromosome 1 affecting fasting glucose levels with one (*Nidd5/of*) around D1Rat46 that explains 7.8% of phenotypic variance, and the other (*Nidd6/of*) around *D1Rat90* that explains 10.7% of phenotypic variance. A chromosome 5 gene (*Nidd7/of*), near *D5Mit11*, showed a linkage to fasting glucose levels. A chromosome 9 locus (*Nidd8/of*), near *D9Rat21*, showed a linkage to glucose levels 90 min after the glucose challenge. A locus (*Nidd9/of*), linked to *D12Mgh5* on chromosome 12, designates a locus for fasting glucose levels. A chromosome 14 QTL (*Nidd10/of*) linked to *CCKAR* (gene encoding cholecystokinin type A receptor) had a significant effect on glucose levels 60 min after glucose challenge. The region (*Nidd11/of*) between *D16Wox7* and *D16Rat13* on chromosome 16 was linked to glucose levels 60 min after the challenge. 

 Furthermore, we detected three novel QTLs (*Nidd12-14/of*) on chromosomes 5, 7, and 17 [33] (Table 1), by using the MapQTL computer program which is applied for the MQM-mapping based on multiple-QTL models and reported to be more powerful than interval mapping [39–41]. We found evidence for a novel QTL near *D5Mgh22* on chromosome 5 (*Nidd12/of*), which was linked to the AUC for the entire glucose challenge. A gene on chromosome 7 (*Nidd13/of*), near *D7Rat35*, showed a linkage to fasting glucose levels. A QTL on chromosome 17 (*Nidd14/of*), near *At1*, was linked to the AUC for the entire glucose challenge.

 Interestingly, *Nidd8/of*, *Nidd9/of*, and *Nidd13/of*, as well as *Nidd3/of*, exhibited heterosis. Further, there were epistasis between *Nidd1/of* and *Nidd10/of*, between *Nidd2/of* and *Nidd8/of*, and between *Nidd2/of* and *Nidd14/of*, as well as between *Nidd1/of* and *Nidd2/of*.

 The *Nidd4*, *5*, *6*, *7*, *9*, *13*, and *14/of* affected the fasting glucose levels (Table 1) and, together, explained 55.6% of the total phenotypic variance or 70.4% of the genetic variance in fasting glucose levels in the F_2_, in a multiple QTL model. Furthermore, the *Nidd1*, *2*, *3*, *6*, *8*, *10*, *11*, *12,* and *14/of* had effects on the postprandial glucose levels (Table 1) and, together, explained 54.6–78.8% of the total phenotypic variance or 71.8–93.8% of the genetic variance in postprandial glucose levels, depending upon the time of measurements, in the F_2_.

 Previous studies have identified NIDDM susceptibility loci on chromosomes 1, 2, 4, 5, 8, 10, and 17 in the GK rat [19, 20] and on chromosomes 1, 12, and 16 in the obese *Lepr* ^*fa*^/*Lepr* ^*fa*^ WKY13 M rat [42]. The *Nidd/gk1* and *Niddm1* identified on chromosome 1 in the GK rat, respectively, may be homologous to the *Nidd5* and *6/of* in the OLETF rat (Table 2). Further, the *Nidd/gk6* identified on chromosome 17 in the GK rat may be homologous to the *Nidd14/of* in the OLETF rat (Table 2). On the other hand, the *Nidd/gk5* identified on chromosome 8 in the GK rat, the *Nidd/gk4* on chromosome 5 in the GK rat, the QTL identified on chromosome 12 in the *Lepr* ^*fa*^/*Lepr* ^*fa*^ WKY13 M rat, and the QTL identified on chromosome 16 in the *Lepr* ^*fa*^/*Lepr* ^*fa*^ WKY13 M rat would not appear to correspond to the *Nidd3*, *7* and *12*, *9*, and *11/of* in the OLETF rat, respectively, because the most likely positions for these loci are separated by ~50 cM for chromosome 8, by ~35 cM and ~50 cM for chromosome 5, by ~20 cM for chromosome 12, and by ~40 cM for chromosome 16. Thus, the majority of total 14 loci for NIDDM in the OLETF rat are clearly distinct from NIDDM susceptibility loci in the GK and the *Lepr* ^*fa*^/*Lepr* ^*fa*^ WKY13 M rats reported previously, consistent with genetic heterogeneity of this disease.

## 5. Relationship between NIDDM QTLs and Body Weight

It has been demonstrated that an increase in body weight is an important risk factor for NIDDM development in the OLETF rat [43]. We thus tried to identify QTLs affecting an increase in body weight. We found one major locus responsible for 11.9% of the genetic variance that was linked to *Ppy* on chromosome 10 with a LOD score of 4.36, designated *Bw1/of* [30]. In addition to the *Bw1/of* region, there existed evidence for suggestive linkage of the trait of body weight to the same regions as *Nidd6* and *11/of* that are responsible for glucose levels [32].

 We have categorized these NIDDM QTLs into three types in terms of their relationship with body weight [32, 33] (Table 1). One type (*Nidd6* and *11/of*) represents the gene contributing to NIDDM through an increase in body weight or affecting pleiotropically to both NIDDM and weight increase. Another type (*Nidd1*, *2*, *8*, *9*, *10,* and *12/of*) designates the gene, not linked to body weight, but interacting with body weight for NIDDM development. The other type (*Nidd3*, *4*, *5*, *7*, *13* and *14/of*) includes the gene possessing action independent of body weight for NIDDM development.

## 6. Suggested Positional Candidate Gene

We have mapped the *Nidd1-14/of* to 11, 10, 17, 13, 21, 13, 11, 12, 8, 10, 27, 10, 13, and 16 cM 1-LOD support intervals, respectively [30, 32, 33]. Several interesting candidate genes were located in these rat genomic regions or the syntenic regions in human genome [30, 32, 33]. Physiologically relevant candidate genes suggested are *CA3*, *GAD3,* and *SSTR3* for *Nidd1/of*, *APOA1*, *APOA4* and *KCNJ5* for *Nidd3/of*, *KNG*, *SST,* and *APOD* for *Nidd4/of*, *GANAB*, *TPH*, *SUR* and *KCNJ11* for *Nidd5/of*, *IDE* for *Nidd6/of*, *NEUROD1* for *Nidd8/of*, *TCF1*/*MODY3* for *Nidd9/of*, and *IGF1* for *Nidd13/of* (Table 2). In addition, *NIDDM1*, *IDDM7*, *IDDM12,* and *IDDM13* loci for the *Nidd8/of* and *NIDDM2* locus for the *Nidd9/of* may be defining the same underlying gene as the present QTLs (Table 2). It is particularly interesting that the *CCKAR* maps in the center of the *Nidd10/of* interval, since the *CCKAR* has been reported to be implicated as a mediator of pancreatic growth in response to cholecystokinin and tumorigenesis in the pancreas [44, 45], and since Takiguchi et al. [46] have reported a homozygous DNA deletion in the OLETF *CCKAR* gene which includes the promoter region and the first and second exons, and therefore leads to defect of its expression in the pancreas. Indeed, we showed that a major QTL colocalizing with *CCKAR* influences poor pancreatic proliferation in the OLETF rat [47].

## 7. Conclusion

1. NIDDM and an increase in body weight in the OLETF rat are controlled by multiple genes.
2. There is a strong positive correlation between body weight and the glucose levels in the F_2_ progeny, consistent with the role of obesity as an important factor in NIDDM development in the OLETF rat. However, NIDDM and body weight are almost under different genetic control.
3. Two significant QTLs for NIDDM are located on chromosomes 7 and 14, and twelve suggestive NIDDM QTLs on chromosomes 1, 5, 8, 9, 11, 12, 14, 16, and 17.
4. Epistasis and heterosis of these QTLs are likely to be involved in NIDDM development in the OLETF rat.
5. One significant QTL for body weight is located on chromosome 10. Two NIDDM QTLs on chromosomes 1 and 16 affect body weight, and six NIDDM QTLs on chromosomes 5, 7, 9, 12, and 14 have an interaction with body weight.
6. NIDDM QTLs are categorized into three types in terms of their relationship with body weight.
7. These suggest a higher degree of genetic complexity in NIDDM of the OLETF rat.
8. The mapped loci for NIDDM in the OLETF rat are almost distinct from those in the GK and the *Lepr* ^*fa*^/*Lepr* ^*fa*^ WKY13 M rats, consistent with genetic heterogeneity of this disease.

## Figures and Tables

**Table 1:** NIDDM loci and their actions to increase glucose levels in combination with body weight.

Locus	Mapping method	Chromosome	Trait	LOD score	Action of gene
*Nidd1/of*	Interval	7	Glucose 60 min	4.33	Interaction
*Nidd2/of*	Interval	14	Glucose AUC	5.15	Interaction
*Nidd3/of*	Interval	8	Glucose 30 min	3.06	Direct action
*Nidd4/of*	Interval	11	Fasting glucose	3.70	Direct action
*Nidd5/of*	Interval	1	Fasting glucose	2.82	Direct action
*Nidd6/of*	Interval	1	Glucose 30 min	3.79	Pleiotropic
*Nidd7/of*	Interval	5	Fasting glucose	3.54	Direct action
*Nidd8/of*	Interval	9	Glucose 90 min	3.87	Interaction
*Nidd9/of*	Interval	12	Fasting glucose	3.72	Interaction
*Nidd10/of*	Interval	14	Glucose 60 min	4.06	Interaction
*Nidd11/of*	Interval	16	Glucose 60 min	3.54	Pleiotropic
*Nidd12/of*	MQM	5	Glucose AUC	3.98	Interaction
*Nidd13/of*	MQM	7	Fasting glucose	3.90	Direct action
*Nidd14/of*	MQM	17	Glucose AUC	4.20	Direct action

**Table 2:** Physiologically relevant positional candidate genes and QTLs for the *Nidd1-14/of*.

Locus	Genes	QTLs
*Nidd1/of*	*CA3*, *GAD3*, *SSTR3 *	—
*Nidd2/of*	—	—
*Nidd3/of*	*APOA1*, *APOA4*, *KCNJ5 *	—
*Nidd4/of*	*KNG*, *SST*, *APOD *	—
*Nidd5/of*	*GANAB*, *TPH*, *SUR*, *KCNJ11 *	*Nidd/gk1*
*Nidd6/of*	*IDE*	*Niddm1*
*Nidd7/of*	—	—
*Nidd8/of*	*NEUROD1*	*NIDDM1*, *IDDM7*, *IDDM12*, *IDDM13 *
*Nidd9/of*	*TCF1*/*MODY3 *	*NIDDM2*
*Nidd10/of*	*CCKAR*	—
*Nidd11/of*	—	—
*Nidd12/of*	—	—
*Nidd13/of*	*IGF1*	—
*Nidd14/of*	—	*Nidd/gk6*

^—^Not detected.
